# Hidden failure modes of large language models in healthcare-associated infection surveillance: a structured evaluation using NHSN definitions

**DOI:** 10.1017/ice.2026.10444

**Published:** 2026-06

**Authors:** Mamdooh Alzyood, Alfred Veldhuis, Hayley Stevenson, Samina Sheikh

**Affiliations:** 1 Faculty of Health Science, and Technology, School of Psychology, Social Work and Public Health, Oxford Brookes University Faculty of Health and Life Sciences, Oxford, UK; 2 Warwickshire College Group/ WCG: Royal Leamington Spa College, UK; 3 London Borough of Sutton, UK

## Abstract

**Background:**

Large language models (LLMs) are increasingly explored for healthcare-associated infection (HAI) surveillance, but their reliability in applying formal National Healthcare Safety Network (NHSN) definitions is not well characterized. This study evaluates GPT-5.1 Thinking’s accuracy and rationales in classifying NHSN-defined infections.

**Methods:**

Seventy synthesized case vignettes containing complete, organized clinical data representing five NHSN infection types, including complex edge cases, were assessed using 2025 NHSN surveillance definitions. GPT-5.1 Thinking classified cases under three prompting strategies: standard, structured, and constrained. Quantitative accuracy metrics and qualitative inductive content analysis of rationales and failure modes were performed.

**Results:**

Overall accuracy across 210 classifications improved from 78.6% (standard prompt) to 88.6% (structured) and 95.7% (constrained). Performance was highest for infections with clear anatomical or radiographic criteria (surgical site infections [SSI], ventilator-associated pneumonia [VAP]) and lowest for infections involving complex exclusion rules (central line-associated bloodstream infection [CLABSI], *Clostridioides difficile* infection [CDI]). Constrained prompting enhanced adherence to NHSN rules but did not eliminate errors in hierarchical exclusions. Content analysis identified three recurrent failure categories: prioritization of clinical plausibility over surveillance logic, failure to apply quantitative and temporal thresholds, and errors in hierarchical source attribution.

**Conclusion:**

GPT-5.1 Thinking shows potential to support infection surveillance under strict constraints but exhibits systematic limitations, including overreliance on clinical intuition and difficulty with complex exclusion pathways. Currently, LLMs are unsuitable for autonomous NHSN classification but may serve as supervised decision-support tools with robust human oversight. Further development is needed to enhance LLMs’ ability to synthesize surveillance definitions and complex situational characteristics critical for effective HAI surveillance, though fully autonomous deployment would require further validation. These findings are based on synthetic data that may differ from real-world clinical data in ways likely to overestimate the accuracy of these tools.

## Introduction

LLMs, especially advanced ones like GPT-5.1, are being explored to automate HAI surveillance^1,2,3^ Research indicates LLMs can streamline workflows, reduce data processing time, and may improve consistency in applying national surveillance definitions.^4,5^ Early studies report promising results in identifying CLABSI, CAUTI, SSI, CDI, and VAP, with potential efficiency and accuracy gains.^4,6,7^

However, performance under NHSN’s strict criteria remains unclear. Prior studies often use simplified cases, limiting understanding of model behavior when data are incomplete, temporal or quantitative thresholds apply, or colonization must be distinguished from infection.^8–10^ NHSN criteria depend on rigid rules, such as toxin-based CDI algorithms, colony-forming unit (CFU) thresholds, device-day windows, and hierarchical secondary bloodstream infection (BSI) attribution. Few studies assess LLM responses to conflicting diagnostic data,^11^ and real-world evidence shows frequent misapplication of NHSN requirements; for example, GPT-4.0 reported promising sensitivity but limited specificity/attribution challenges for CLABSI and misattributed secondary BSIs.^12^ Broader LLM reasoning limitations, including inflexibility and unsupported inference, have been noted^10^ but not systematically studied within surveillance frameworks requiring definitional precision.

This study evaluates GPT-5.1 Thinking’s application of NHSN surveillance definitions across five HAI categories using 70 synthesized vignettes containing complete, organized clinical information under three prompting strategies, assessing quantitative accuracy and rationales provided for misclassifications. These aims align with the 4Ps framework for responsible AI in infection surveillance, emphasizing precision, partnership, practice, and people for safe AI integration.^13^

## Methods

### Study design

This cross-sectional evaluation assessed GPT-5.1 Thinking’s performance in determining whether case vignettes met the official NHSN 2025 surveillance definitions for five major HAI types: CLABSI, CAUTI, CDI, SSI, and VAP.^14^ A convergent mixed methods design was employed.^15^ The quantitative strand assessed accuracy across HAI categories and prompting strategies using predefined gold-standard outcomes based strictly on NHSN rules. The qualitative strand applied inductive qualitative content analysis to rationales from misclassified vignettes (*n* = 26) to explain quantitative error patterns,^16^ coding segments where the model’s justifications diverged from NHSN rules. Codes were derived inductively from the data, then grouped into categories through iterative comparison. A full list of codes, case identifiers, and assigned categories is provided in Supplementary File 1.

### Case vignette development

Seventy scenario-based case vignettes were developed following established methodology for evaluating decision support systems.^17^ The vignette set comprised 28 positive cases meeting full NHSN criteria and 42 negative cases where criteria were not met, ensuring evaluation of both sensitivity and specificity. The set included CLABSI (*n* = 17; 6 positive, 11 negative), CAUTI (*n* = 14; 4 positive, 10 negative), CDI (*n* = 14; 6 positive, 8 negative), SSI (*n* = 12; 6 positive, 6 negative), and VAP (*n* = 13; 6 positive, 7 negative). The first 50 vignettes (C1-C50) reflected core surveillance situations; the remaining 20 (C51-C70) were high-complexity edge cases designed to stress-test hierarchical rules, device-day windows, secondary BSI attribution, and quantitative thresholds.

SSI vignettes included cases with objective findings (purulent drainage, positive cultures, imaging-confirmed abscesses) and cases requiring differentiation from non-infectious postoperative complications (seromas, hematomas). All vignettes and gold-standard classifications are provided in Supplementary File 2. Importantly, all vignettes contained complete, well-organized clinical data presented in a structured format. Real-world clinical documentation is typically more fragmented, incomplete, and narratively complex; accordingly, results from these vignettes are likely to overestimate the accuracy achievable in routine clinical practice.

### Gold-standard NHSN classifications

Gold-standard classifications were assigned by the lead author (MA, Infection Prevention and Control Consultant) and independently verified by two co-authors with expertise in public health and infection surveillance. Discrepancies were resolved through consensus discussion referencing the 2025 NHSN Patient Safety Component Manual (PSCM) criteria.^14^ NHSN surveillance applies strict binary criteria: an infection is counted only if all required criteria are clearly met.

### LLM tested

GPT-5.1 Thinking (OpenAI) was selected as a current-generation model with advanced reasoning capabilities. OpenAI describes GPT-5.1 Thinking as intended for more complex tasks/more persistent reasoning,^18^ making it appropriate for structured classification tasks. All classifications were generated in October 2025.

### Prompting strategies

Three prompting strategies were used. The 2025 NHSN PSCM definitions for each HAI type were uploaded to GPT-5.1 Thinking as reference documents prior to case evaluation. The standard prompt requested a direct Yes/No classification with brief justification. The structured prompt required stepwise reasoning: summarize key clinical findings, identify criteria met and not met, then provide final classification. The constrained prompt enforced explicit rule checking with strict Yes/No determination. All prompts requested written rationales. Full prompt text is provided in Supplementary File 2.

### Evaluation metrics

**Figure 1.**
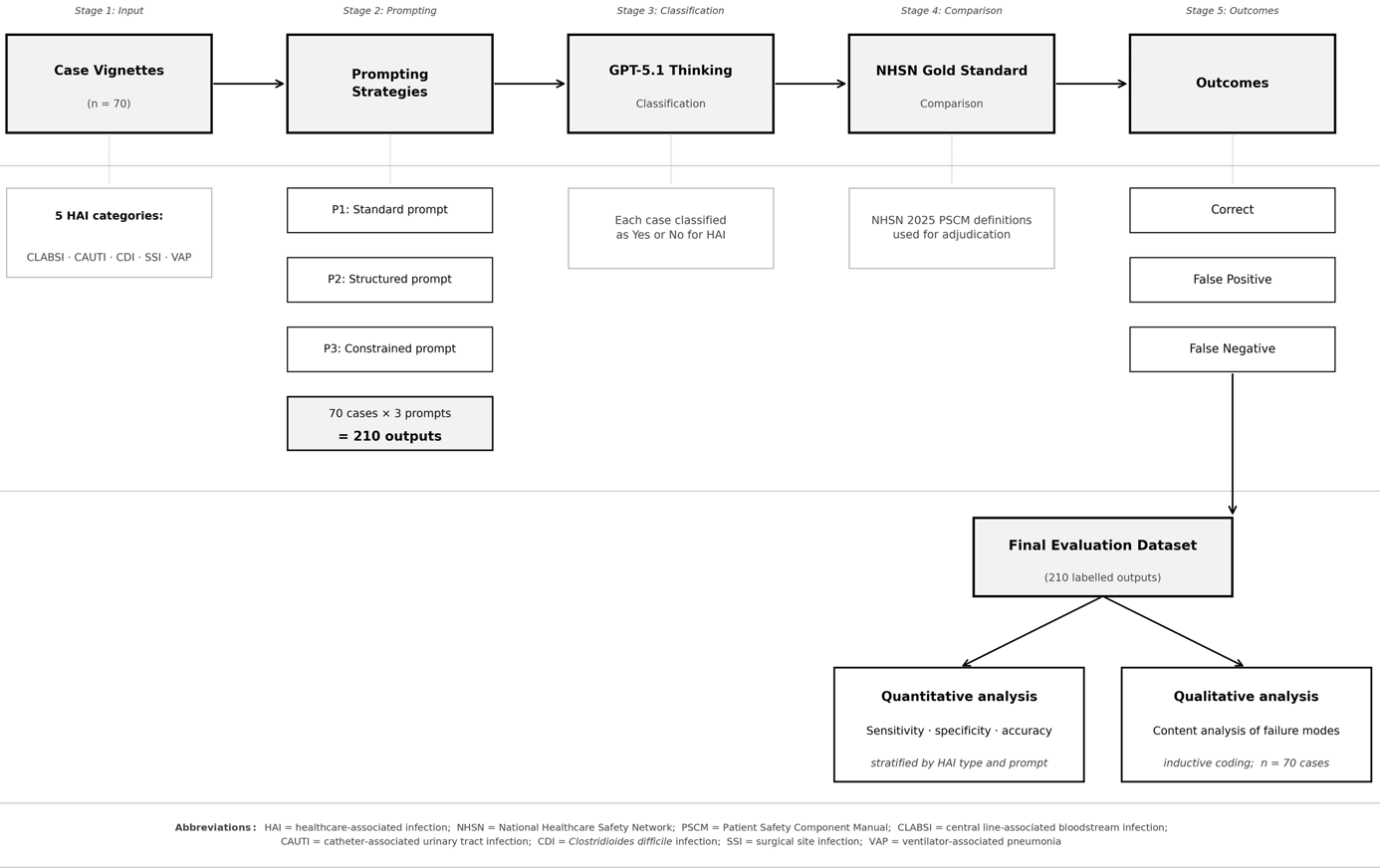
Figure 1 long description. Stepwise evaluation workflow showing case input, prompting strategies, and classification output. Each vignette was evaluated under standard, structured, and constrained prompts. Outputs were compared with gold-standard NHSN 2025 classifications to generate quantitative accuracy metrics. Misclassified vignettes were subject to inductive content analysis. HAI, healthcare-associated infection; NHSN, National Healthcare Safety Network; CLABSI, central line-associated bloodstream infection; CAUTI, catheter-associated urinary tract infection; CDI, *Clostridioides difficile* infection; SSI, surgical site infection; VAP, ventilator-associated pneumonia.

Each of the 70 vignettes was presented once under each prompting strategy, generating 210 classifications (Figure 1 shows the evaluation workflow). Performance was evaluated using accuracy, sensitivity, specificity, false positive rate, and false negative rate^19^. Cochran’s Q test assessed differences across strategies, with post hoc pairwise exact McNemar tests. HAI-specific comparisons were summarized descriptively due to limited cell counts.

### Ethical considerations

This study did not involve human participants or real patient data. All case vignettes were created de novo, were entirely synthetic, and contained no identifiable information. Ethical approval and informed consent were therefore not required, and the work was considered exempt from ethical review.

## Results

Seventy synthetic case vignettes were evaluated, comprising 28 NHSN-positive cases and 42 NHSN-negative cases. The vignette set covered five NHSN HAI categories: CLABSI (*n* = 17; 6 positive, 11 negative), CAUTI (*n* = 14; 4 positive, 10 negative), CDI (*n* = 14; 6 positive, 8 negative), SSI (*n* = 12; 6 positive, 6 negative), and VAP (*n* = 13; 6 positive, 7 negative). Each vignette was assessed once under each prompting strategy (standard, structured, constrained), yielding 210 case-prompt classifications for quantitative analysis (Figure 1).

### Quantitative findings

Overall accuracy differed significantly by prompt structure (Cochran’s Q(2) = 18.2, *P* < .001). The constrained (95.7%) and structured (88.6%) prompts showed higher accuracy than the standard (78.6%) prompt (McNemar *P* = .00049 and *P* = .0156, respectively). Sensitivity for detecting NHSN-qualifying HAI events remained high across all prompts (96.4–100%). Accuracy was not significantly different between structured and constrained prompting (McNemar *P* = .0625). The false-positive rate fell from 33.3% under standard prompting to 7.1% with constrained prompting, indicating progressively tighter adherence to NHSN rule thresholds.

### Accuracy by infection category

As HAI-specific analyses were underpowered for formal statistical testing, these differences should be interpreted descriptively. SSI showed the highest observed accuracy (97.2%), followed by CAUTI (92.9%) and VAP (89.7%). Accuracy was numerically lower for CDI (81.0%) and CLABSI (80.4%), with errors reflecting difficulty applying hierarchical exclusions and distinguishing colonization from infection (Table 1).

**Table 1.** Descriptive accuracy across HAI categories Table 1 long description.

HAI type	*n*	Correct	Accuracy	FP	FN	Key error patterns
SSI	36	35	97.2%	1	0	Misclassified non-infectious seroma as superficial SSI
VAP	39	35	89.7%	4	0	Overcalled VAP with equivocal radiology; ignored postextubation timing window
CAUTI	42	39	92.9%	3	0	Ignored CFU threshold (<10^5^); classified despite ineligible mixed flora
CDI	42	34	81.0%	7	1	Equated NAAT positivity with infection despite negative toxin and alternative causes
CLABSI	51	41	80.4%	10	0	Failed MBI-LCBI exclusion; misattributed secondary BSI as primary CLABSI
**TOTAL**	**210**	**184**	**87.6%**	**25**	**1**	

*Note: n*, case-prompt observations (cases × 3 prompting strategies); FP, false positive; FN, false negative.

## Qualitative findings: Content analysis of failure modes

Analysis of the 26 misclassified vignettes identified three categories capturing recurring ways in which GPT-5.1 Thinking failed to apply NHSN criteria. Two additional cases (C59, C68) produced errors that did not fit the primary categories and are detailed in Supplementary File 1.

### Category 1: Clinical plausibility overriding surveillance rules

The model consistently inferred infection when clinically plausible, even when mandatory NHSN criteria were not met. In CDI cases, positive nucleic acid amplification test (NAAT) results overrode toxin requirements and alternative explanations. In Case 58, the model equated NAAT positivity with CDI despite a negative toxin assay and high-dose magnesium use providing a clear alternative explanation for diarrhea; under structured prompting it acknowledged the magnesium use but still classified CDI. Case 67 showed the same pattern: only two loose stools were documented (below the NHSN threshold of three) following enteral feed adjustment, yet CDI was assigned under standard and structured prompts. In VAP cases, equivocal radiology was similarly dismissed. In Case 60, the chest radiograph reported no definite new consolidation, yet the model classified VAP across standard and structured prompts, recognizing the equivocal findings but still concluding infection was present. The most persistent error involved mucosal barrier injury laboratory-confirmed bloodstream infection (MBI-LCBI) exclusions. In Case 61, neutropenia (absolute neutrophil count 300 cells/μL), severe mucositis, and *Enterococcus faecium* bacteremia met explicit MBI-LCBI criteria, yet the model classified it as CLABSI across all three prompting strategies, representing the most prompt-resistant error observed.

### Category 2: Inconsistent application of quantitative thresholds and timing rules

The model treated numerical thresholds and timing windows as flexible guidelines rather than fixed definitional criteria. For CAUTI, Case 57 involved a urine culture growing 8 × 10^4^ CFU/mL (below the NHSN minimum of 10^5^ CFU/mL), yet CAUTI was assigned under standard prompting. Case 65 showed identical behavior, with a urine culture of 4 × 10^4^ CFU/mL classified as CAUTI, reflecting a pattern of assuming that any combination of bacteriuria, catheter, and symptoms equates to CAUTI regardless of quantitative thresholds. For CLABSI, Case 64 involved a single positive blood culture bottle growing coagulase-negative staphylococci, a known skin contaminant requiring two positive cultures under NHSN rules; the standard prompt classified this as CLABSI despite the single bottle. Temporal rules proved similarly problematic. In Case 63, a central line was removed three days before bacteremia onset, exceeding the two-day postremoval window; the model overlooked the removal timing under standard prompting and, under structured prompting, described the chronology but still concluded CLABSI. In Case 69, pneumonia developed three days after extubation, outside the two-day ventilator window for VAP; the model classified this as VAP on clinical grounds without applying the timing rule, and structured prompting described the extubation timing but retained the VAP classification. These errors indicate that the model recognizes relevant temporal and quantitative information but does not apply it as a mandatory constraint. All errors in this category were resolved by constrained prompting.

### Category 3: Hierarchical attribution errors

The model struggled to apply NHSN’s secondary BSI attribution rules, which require linking bacteremia to a primary infection site when a matching organism is identified elsewhere. In Case 62, a patient with lobar consolidation had *Streptococcus pneumoniae* in both blood and sputum cultures. NHSN rules require attribution to the respiratory source as secondary BSI, excluding CLABSI; however, the model applied the heuristic that any bacteremia with a central line present indicates line infection and classified it as CLABSI under standard prompting. Case 51 showed the same failure: *Klebsiella pneumoniae* bacteremia with abdominal pain and imaging findings attributable to an intra-abdominal source was misclassified as CLABSI across all prompts. Case 66 illustrated a related error: CAUTI was assigned based on pyuria and bacteriuria, despite the urine culture growing mixed flora (ineligible under NHSN) and new pulmonary infiltrates indicating pneumonia as the primary source. The MBI-LCBI exclusion pathway exhibited the most persistent failures, with Case 61 already described under Category 1. These findings indicate that hierarchical source attribution logic remains beyond the reliable capabilities of this model under the tested prompting strategies.

## Discussion

This evaluation suggests GPT-5.1 Thinking performance in NHSN surveillance varied with the complexity of the underlying definition. The model achieved higher accuracy for HAIs governed by straightforward criteria (SSI and VAP), while reliability declined for definitions requiring complex hierarchical source attribution or strict temporal thresholds (CDI and CLABSI). Consistent with prior work, these findings indicate that LLMs tend to overcall HAIs: they are overly sensitive and insufficiently specific. This pattern has direct clinical implications, as artificially inflated HAI rates are highly undesirable for hospitals from both quality and regulatory perspectives.

These observations are consistent with evidence that surveillance definitions requiring greater interpretive judgment are inherently more challenging to apply consistently.^20^ A 2024 systematic review reported that NLP models achieved up to 99% specificity in pneumonia detection, whereas GPT-4 demonstrated only 59% diagnostic accuracy for BSI.^21^ Similar patterns appear in studies showing LLM performance varies with task complexity.^22^ Our findings demonstrate that accurate clinical interpretation does not translate into correct application of NHSN rules when classifications depend on thresholds, timing windows, or hierarchical exclusions.

Our findings warrant contextualization against recent real-world evaluations. Rodriguez-Nava et al reported LLMs could rapidly assess CLABSI with high sensitivity using clinical notes at Stanford, viewing LLMs as promising tools to assist rather than replace human review.^12^ Morgan et al noted that some borderline cases may reasonably be classified either way, making definitive determinations less straightforward for infections with subjective elements.^3^ Several methodological factors may explain apparent discrepancies with our results. First, our evaluation used synthetic vignettes containing complete information, whereas real-world notes may contain ambiguities that paradoxically simplify classification. Second, we tested a single model with standardized prompts, whereas institutional implementations may involve fine-tuned approaches. Third, our focus on edge cases deliberately stressed failure modes that may occur less frequently in routine surveillance populations.

The errors in CDI and CLABSI classifications suggest difficulty applying multi-step NHSN logic. However, some NHSN criteria, particularly source attribution in CLABSI, contain inherent subjectivity that challenges both human and automated adjudication.^3^ The persistent tendency to overcall infection when microbiological signals are present, combined with failures in hierarchical attribution and temporal logic, suggests that unmonitored deployment risks inflating HAI rates.

## Implications for practice and research

Currently, GPT-5.1 Thinking cannot function as an autonomous HAI surveillance tool. However, its high sensitivity indicates potential utility for identifying cases warranting review, while infection preventionists retain responsibility for final decisions. This aligns with the Partnership pillar of the 4Ps framework.^13^

Three practical implications emerge. First, organizations may benefit from prompts aligned with surveillance rules, given that accuracy increased from 78.6% to 95.7% with constrained prompting. Second, developing AI literacy within infection prevention teams could help staff interpret model outputs effectively. Third, governance processes should incorporate periodic auditing to identify performance drift. Future research should examine whether these error patterns appear across different LLM architectures and explore hybrid approaches combining language models with deterministic rule engines.

## Strengths and limitations

This is the first systematic evaluation of GPT-5.1 Thinking across the full range of NHSN surveillance definitions using the 2025 PSCM. The 70 vignettes captured definitional nuances including secondary BSI attribution, MBI-LCBI exclusions, and quantitative thresholds. Evaluating three prompting strategies revealed performance changes with prompt design.

Limitations include use of standardized vignettes that lack the complexity of real-world documentation. We evaluated one model at a single time point; results may not generalize to other LLMs or future versions. The model was not connected to live electronic health records; challenges of automated data extraction were not assessed. As NHSN definitions evolve, our findings reflect 2025 PSCM criteria. Furthermore, the ability to detect specific HAI types may be influenced by the complete and organized nature of information provided in the clinical vignettes; real-world detection is likely to be lower as more information will typically be missing or ambiguous. Thus, the estimates of accuracy presented in this study represent an upper bound of what these tools may achieve in practice. Taken together, these findings suggest that LLMs of this kind require human oversight and are not yet ready for fully autonomous deployment in HAI surveillance.

## Conclusion

GPT-5.1 Thinking showed improved performance with constrained prompting closely aligned with NHSN definitions. Classifications were more reliable for infections defined by clear, observable criteria and less reliable for infections depending on complex exclusion rules, timing windows, and hierarchical source attribution. Persistent difficulties in applying numerical thresholds and source attribution suggest these weaknesses may reflect limitations in current LLM reasoning beyond simple prompt design issues. Our findings indicate that fully autonomous NHSN classification using GPT-5.1 Thinking with these prompting strategies showed limitations that would require resolution before unsupervised deployment. Whether different models, prompting approaches, or validated commercial products might achieve acceptable autonomous performance remains an open question.

## Supporting information

Alzyood et al. supplementary material 1 Alzyood et al. supplementary material

Alzyood et al. supplementary material 2 Alzyood et al. supplementary material

## Supplementary material

The supplementary material for this article can be found at https://doi.org/10.1017/ice.2026.10444.

## Figure 1 Long description

The flowchart depicts a stepwise evaluation workflow for healthcare-associated infection surveillance using large language models. The process begins with case vignettes, which are input into the system. These vignettes are categorized into five healthcare-associated infection categories: CLABSI, CAUTI, CDI, SSI, and VAP. The next stage involves prompting strategies, which include a standard prompt, a structured prompt, and a constrained prompt. Each of the 70 cases is evaluated under these three prompts, resulting in 210 outputs. These outputs are then classified by GPT-5.1 Thinking, where each case is classified as Yes or No for healthcare-associated infection. The classified outputs are compared with the NHSN Gold Standard using the NHSN 2025 PSCM definitions for adjudication. The outcomes are categorized as correct, false positive, or false negative. The final evaluation dataset consists of 210 labeled outputs, which undergo quantitative analysis to determine sensitivity, specificity, and accuracy, stratified by healthcare-associated infection type and prompt. Additionally, qualitative analysis involves content analysis of failure modes through inductive coding of the 70 cases.

Navigate back to Figure 1.

## Table 1 Long description

The table presents data on descriptive accuracy across various healthcare-associated infection (HAI) categories. It includes columns for HAI type, number of cases (n), number of correct identifications, accuracy percentage, false positives (FP), false negatives (FN), and key error patterns. The table has six rows and seven columns. SSI shows the highest accuracy at 97.2%, followed by CAUTI at 92.9% and VAP at 89.7%. CDI and CLABSI have lower accuracy rates at 81.0% and 80.4%, respectively. Key error patterns include misclassification of non-infectious seroma as superficial SSI, overcalled VAP with equivocal radiology, ignored CFU threshold for CAUTI, equating NAAT positivity with infection for CDI, and failed MBI-LCBI exclusion for CLABSI.

Navigate back to Table 1.
