# Supplementary material for: Hidden failure modes of large language models in healthcare-associated infection surveillance: a structured evaluation using NHSN definitions
Source: Infect Control Hosp Epidemiol. 2026 Apr 6;47(6):568–73. doi: 10.1017/ice.2026.10444 (PMC13216792; doi:10.1017/ice.2026.10444)
Supplement: Alzyood et al. supplementary material 1 — Alzyood et al. supplementary material [file S0899823X26104449sup001.docx]

Supplementary File 1

Categories and failure-mode codes used in the qualitative analysis

**Overview**: Seventy clinical vignettes (C1-C70) were evaluated by GPT-5.1 Thinking using three prompting strategies (Standard, Structured, Constrained). Of these, 44 cases (C1-C50, C52, C54-C56, C70) were correctly classified across all prompts and are not included in this qualitative analysis. The remaining 15 cases (C51, C53, C57-C69) produced 26 misclassification instances, which form the basis of this content analysis. Cases C51, C53, and C61 showed errors resistant to all prompting strategies; the remainder were corrected by Structured and/or Constrained prompts.

**Table S1.** Content analysis coding framework: From initial codes to final categories derived from analysis of 26 misclassified vignettes across three prompting strategies.

| **Case** | **Initial Code** | **Failure Mode** | **Representative Data Extract** | **Prompt Response Pattern** |
| --- | --- | --- | --- | --- |
| **Category 1: Clinical plausibility overriding surveillance rules** | | | | |
| **C53** | NAAT-over-toxin | Misclassification: did not apply toxin requirement | *"GPT misclassified NAAT+/toxin− as CDI". Model equated positive molecular test with infection despite negative toxin assay* | Error persisted across all three prompts (Standard, Structured, Constrained) |
| **C58** | NAAT-over-toxin with alternative-cause-override | Definitional mismatch: ignored toxin and alternative explanation | *"Equated NAAT positivity with CDI despite alternative cause". High-dose magnesium use provided clear alternative explanation for diarrhoea* | Standard & Structured: Error; Constrained: Correct (applied toxin requirement and alternative cause exclusion) |
| **C67** | PCR-driven-classification | Overcalling CDI: did not enforce stool count or alternative cause exclusion | *"Interpreted NAAT positivity as CDI despite insufficient stools and clear alternative cause". Only 2 loose stools (threshold: ≥3) with enteral feed adjustment* | Standard & Structured: Error ("described feed-related diarrhoea but still concluded CDI"); Constrained: Correct |
| **C60** | Radiograph-override | Definition mismatch: did not require clear radiological progression | *"Classified clinically as VAP despite lack of definitive new infiltrate". CXR reported "no definite new consolidation" yet model classified VAP* | Standard & Structured: Error ("recognised equivocal radiology but still labelled as VAP"); Constrained: Correct |
| **C61** | MBI-LCBI-ignored | Misclassification: MBI-LCBI mislabelled as CLABSI | *"GPT incorrectly classified an MBI-LCBI as CLABSI". Neutropenia (ANC 300), severe mucositis, and E. faecium met explicit MBI-LCBI criteria* | Error persisted across all three prompts, most resistant failure mode |
| **Category 2: Inconsistent application of quantitative thresholds and timing rules** | | | | |
| **C57** | CFU-threshold-ignored | Threshold error: ignored CFU cut-off | *"GPT overcalled CAUTI despite CFU <10⁵ and limited symptoms". Urine culture 8×10⁴ CFU/mL E. coli (NHSN minimum: 10⁵ CFU/mL)* | Standard: Error; Structured & Constrained: Correct (explicitly considered CFU threshold) |
| **C65** | CFU-threshold-ignored | Threshold error: ignored 10⁵ CFU requirement | *"Assumed any bacteriuria + catheter + symptoms = CAUTI". Urine culture 4×10⁴ CFU/mL (<10⁵ threshold)* | Standard: Error; Structured & Constrained: Correct (explicitly linked decision to CFU threshold) |
| **C64** | Culture-count-threshold | Threshold error: did not require ≥2 positive cultures | *"Called CLABSI on a single CNS bottle". Single positive bottle with CoNS, repeat cultures negative; meets contaminant definition* | Standard: Error; Structured & Constrained: Correct (recognised CNS as contaminant) |
| **C63** | Device-timing-rule-violated | Timing rule error: ignored ≤2-day device window | *"Overlooked removal timing and treated as CLABSI". Central line removed 3 days before bacteraemia onset (exceeds 2-day post-removal window)* | Standard: Error; Structured: Error ("described chronology but still concluded CLABSI"); Constrained: Correct |
| **C69** | Ventilator-timing-rule-violated | Timing rule error: ignored post-extubation window | *"Classified as VAP on clinical grounds without checking ventilator timing". Pneumonia developed 3 days post-extubation (outside 2-day ventilator window)* | Standard: Error; Structured: Error ("described extubation timing but still labelled VAP"); Constrained: Correct |
| **Category 3: Hierarchical attribution errors** | | | | |
| **C51** | Secondary-BSI-misattribution | Misclassification: secondary BSI mislabelled as CLABSI | *"GPT incorrectly classified a secondary BSI as CLABSI". K. pneumoniae bacteraemia with abdominal symptoms; BSI attributable to intra-abdominal source* | Error persisted across all three prompts |
| **C62** | Pneumonia-secondary-BSI-ignored | Incorrect source attribution: secondary BSI mislabelled as CLABSI | *"GPT assumed central line + BSI = CLABSI despite clear pneumonia". S. pneumoniae in blood matched sputum with lobar consolidation on CXR* | Standard: Error; Structured & Constrained: Correct (recognised pneumonia as primary site) |
| **C66** | Alternative-source-ignored | Definition/organism error: used ineligible culture and ignored alternative source | *"Labelled CAUTI based on pyuria and bacteriuria despite mixed flora and pneumonia". Mixed flora not eligible for CAUTI; new infiltrate indicates pneumonia* | Standard: Error; Structured & Constrained: Correct (identified mixed flora as ineligible) |
| **Additional failure modes** | | | | |
| **C59** | Non-infectious-finding-misclassified | Overcalling SSI: misclassified non-infectious seroma | *"Interpreted seroma and erythema as superficial SSI". No purulence, no culture, imaging suggested seroma; symptoms resolved without antibiotics* | Standard: Error; Structured & Constrained: Correct (distinguished seroma from SSI) |
| **C68** | Toxin-rule-under-applied | Misinterpretation of NHSN: over-weighted alternative cause against toxin | *"Incorrectly dismissed CDI because of laxative use despite toxin positivity". Positive toxin meets CDI criteria regardless of laxative context* | Standard: Error (false negative); Structured & Constrained: Correct |

**Table S2.** Summary of categories, codes, and error persistence across prompting strategies

| **Category** | **Constituent Codes** | **Cases (n)** | **Prompt-Resistant Errors** |
| --- | --- | --- | --- |
| **Category 1:** Clinical plausibility overriding surveillance rules | NAAT-over-toxin; Alternative-cause-override; PCR-driven-classification; Radiograph-override; MBI-LCBI-ignored | 5 cases (C53, C58, C60, C61, C67) | C53, C61 (persisted across all prompts) |
| **Category 2:** Inconsistent use of quantitative thresholds and timing rules | CFU-threshold-ignored; Culture-count-threshold; Device-timing-rule-violated; Ventilator-timing-rule-violated | 5 cases (C57, C63, C64, C65, C69) | None (all corrected by Constrained prompt) |
| **Category 3:** Hierarchical attribution errors | Secondary-BSI-misattribution; Pneumonia-secondary-BSI-ignored; Alternative-source-ignored | 3 cases (C51, C62, C66) | C51 (persisted across all prompts) |
| **Additional failure modes** | Non-infectious-finding-misclassified; Toxin-rule-under-applied | 2 cases (C59, C68) | None |

**Abbreviations:** BSI, bloodstream infection; CAUTI, catheter-associated urinary tract infection; CDI, Clostridioides difficile infection; CFU, colony-forming units; CLABSI, central line-associated bloodstream infection; CNS/CoNS, coagulase-negative staphylococci; CXR, chest X-ray; MBI-LCBI, mucosal barrier injury laboratory-confirmed bloodstream infection; NAAT, nucleic acid amplification test; NHSN, National Healthcare Safety Network; SSI, surgical site infection; VAP, ventilator-associated pneumonia.

**Note:** Analysis followed an inductive content analysis approach (Elo S, Kyngäs H. J Adv Nurs 2008;62:107-15). Initial codes were generated inductively from the model's written rationales and failure mode descriptions across 26 misclassified vignettes (15 unique cases × up to 3 prompting strategies). Codes were iteratively refined and grouped into categories through constant comparison. Final categories represent patterns of systematic reasoning failures rather than isolated errors.
