# Supplementary material for: Hidden failure modes of large language models in healthcare-associated infection surveillance: a structured evaluation using NHSN definitions
Source: Infect Control Hosp Epidemiol. 2026 Apr 6;47(6):568–73. doi: 10.1017/ice.2026.10444 (PMC13216792; doi:10.1017/ice.2026.10444)
Supplement: Alzyood et al. supplementary material 2 — Alzyood et al. supplementary material [file S0899823X26104449sup002.docx]

| Table S1. Prompting strategies used in the study | | | |
| --- | --- | --- | --- |
| Prompt type | **Prompt wording used in the study** | **Description** | **Intended purpose** |
| Standard | “*Does this patient meet the NHSN definition for [CLABSI/CAUTI/CDI/SSI/VAP]? Provide a Yes/No answer and a brief explanation based only on the information provided*.” | A conversational question requiring a Yes or No classification with brief justification. | Simulates unstructured real-world user interaction. |
| Structured | “*Evaluate this case using the NHSN definition for [HAI type]. Follow these steps: 1. Summarise key clinical findings. 2. Identify which NHSN criteria are met. 3. Identify which criteria are not met. 4. Provide a final classification: Yes, meets the definition OR No, does not meet the definition. Base your decision strictly on NHSN criteria.*” | A stepwise reasoning format requiring summary of findings, mapping to criteria, and a final classification. | Encourages intermediate reasoning and reduces hallucination. |
| Constrained | “*Evaluate this case strictly according to the official NHSN surveillance definition for [CLABSI/CAUTI/CDI/SSI/VAP]. You must follow each step exactly: 1. Extract all relevant objective data points from the vignette (signs, symptoms, timings, device days, culture results, quantitative thresholds). 2. For each required NHSN criterion, state whether it is met, not met, or not documented. 3. If any required element is not met or not documented, classify the case as No. 4. If all required elements are met, classify the case as Yes. 5. Provide only a final Yes or No classification and a brief reference to the NHSN rule elements used, without introducing clinical assumptions beyond the information provided.*” | A rule-based format requiring extraction of objective data, mapping to all definitional elements, and a strict binary output. | Maximises definitional fidelity and reduces interpretive drift. |

| **Table S2**. Seventy scenario-based clinical case vignettes with GPT prompts and responses | | | | | | | | |
| --- | --- | --- | --- | --- | --- | --- | --- | --- |
| **C =Case #** | **Full Case Description** | **HAI type** | **Gold standard NHSN 2025 definitions** | **Reason** | **Prompt type** | **Final classification (Yes/No)** | **Notes** | **Failure mode** |
| C1 | A 67-year-old man with a right internal jugular central line inserted on hospital day 2 develops fever on day 6. Blood cultures taken on day 6 grow methicillin-sensitive *Staphylococcus* *aureus* in two separate sets. No other identifiable source (urine negative, CXR clear, no wounds). | CLABSI | YES - CLABSI | Meets NHSN LCBI-1 definition with no alternative source and central line >2 calendar days. | Standard | Yes | Correct | None |
| C1 |  | CLABSI | YES - CLABSI |  | Structured | Yes | Correct | None |
| C1 |  | CLABSI | YES - CLABSI |  | Constrained | Yes | Correct | None |
|  | | | | | | | | |
| C2 | A 74-year-old woman with a PICC line develops fever on hospital day 5. Blood culture grows *Klebsiella* *pneumoniae*. Chest X-ray shows consolidation; sputum culture grows the same organism. | CLABSI | No | Meets criteria for secondary BSI from pneumonia; NHSN requires that BSI be secondary when a matching organism is found at the primary site. | Standard | No | Correct | None |
| C2 |  | CLABSI | No |  | Structured | No | Correct | None |
| C2 |  | CLABSI | No |  | Constrained | No | Correct | None |
|  | | | | | | | | |
| C3 | A 54-year-old male had a central line placed on day 3. On day 4, he develops fever, and blood cultures grow *Enterococcus* *faecalis*. | CLABSI | No | Central line not in place for >2 calendar days at the time of event, cannot be CLABSI. | Standard | No | Correct | None |
| C3 |  | CLABSI | No |  | Structured | No | Correct | None |
| C3 |  | CLABSI | No |  | Constrained | No | Correct | None |
|  | | | | | | | | |
| C4 | A 48-year-old woman in ICU with a femoral central line in place for 6 days becomes febrile on day 8. Blood cultures grow *Candida* *albicans*. Urine, sputum, and wound cultures are negative. | CLABSI | YES - CLABSI | *Candida* species always qualify for LCBI-1; no alternate source. | Standard | Yes | Correct | None |
| C4 |  | CLABSI | YES - CLABSI |  | Structured | Yes | Correct | None |
| C4 |  | CLABSI | YES - CLABSI |  | Constrained | Yes | Correct | None |
|  | | | | | | | | |
| C5 | A 60-year-old male with a central line in place for 5 days develops fever. Only one blood culture set was collected, growing coagulase-negative *Staphylococcus*. No second confirmatory culture was drawn. | CLABSI | No | CNS requires two positive cultures from separate venipunctures to meet LCBI criteria; otherwise considered a contaminant. | Standard | No | Correct | None |
| C5 |  | CLABSI | No |  | Structured | No | Correct | None |
| C5 |  | CLABSI | No |  | Constrained | No | Correct | None |
|  | | | | | | | | |
| C6 | A 72-year-old man with a central venous catheter in place for 4 days develops fever and rigors on day 7. Two blood culture sets grow *Enterobacter* *cloacae* and *Enterococcus* *faecium* in both bottles. No abdominal, pulmonary, or urinary source identified. | CLABSI | YES - CLABSI | Meets LCBI-1 with polymicrobial growth, no alternative source, line >2 calendar days. | Standard | Yes | Correct | None |
| C6 |  | CLABSI | YES - CLABSI |  | Structured | Yes | Correct | None |
| C6 |  | CLABSI | YES - CLABSI |  | Constrained | Yes | Correct | None |
|  | | | | | | | | |
| C7 | A 62-year-old man with AML undergoing chemotherapy has a central line in place for 10 days. He is severely neutropenic (ANC 100). Blood cultures grow *E. coli*. He has diarrhoea, grade 3 mucositis, and CT abdomen shows colitis. | CLABSI | No | Organism is eligible for MBI pathway; neutropenia + GI symptoms = MBI-LCBI, which does not count as CLABSI according to NHSN. | Standard | No | Correct | None |
| C7 |  | CLABSI | No |  | Structured | No | Correct | None |
| C7 |  | CLABSI | No |  | Constrained | No | Correct | None |
|  | | | | | | | | |
| C8 | A 45-year-old female with a central line in situ for 5 days develops fever. Blood cultures drawn simultaneously from the central line and a peripheral stick both grow Pseudomonas aeruginosa with identical susceptibilities. | CLABSI | YES - CLABSI | Two or more positive cultures with matching organisms, no secondary source, LCBI-1 CLABSI. | Standard | Yes | Correct | None |
| C8 |  | CLABSI | YES - CLABSI |  | Structured | Yes | Correct | None |
| C8 |  | CLABSI | YES - CLABSI |  | Constrained | Yes | Correct | None |
|  | | | | | | | | |
| C9 | A 69-year-old man with a central line for 3 days develops fever. The central-line blood culture grows Staph epidermidis while the peripheral culture grows *Streptococcus* *mitis*. Only one set of each was drawn. | CLABSI | No | No matching organism in ≥2 cultures, does not meet CLABSI criteria; scenario consistent with contamination. | Standard | No | Correct | None |
| C9 |  | CLABSI | No |  | Structured | No | Correct | None |
| C9 |  | CLABSI | No |  | Constrained | No | Correct | None |
|  | | | | | | | | |
| C10 | A 58-year-old male has a central line in place for 7 days. Fever occurs on day 9. Two sets of blood cultures grow *Enterococcus* *faecium* (VRE). Urinalysis and urine culture are negative; abdominal imaging normal. | CLABSI | YES - CLABSI | Matches LCBI-1 with no other primary source after adequate evaluation. | Standard | Yes | Correct | None |
| C10 |  | CLABSI | YES - CLABSI |  | Structured | Yes | Correct | None |
| C10 |  | CLABSI | YES - CLABSI |  | Constrained | Yes | Correct | None |
|  | | | | | | | | |
| C11 | A 76-year-old woman with an indwelling urinary catheter in place for 4 days develops new fever (38.5°C) and suprapubic tenderness. Urine culture grows ≥10⁵ CFU/mL *E. coli*. No other infection source identified. | CAUTI | YES - CAUTI | Catheter in >2 days, meets symptomatic criteria, urine ≥10⁵ CFU, organism eligible. | Standard | Yes | Correct | None |
| C11 |  | CAUTI | YES - CAUTI |  | Structured | Yes | Correct | None |
| C11 |  | CAUTI | YES - CAUTI |  | Constrained | Yes | Correct | None |
|  | | | | | | | | |
| C12 | A 59-year-old man with a Foley catheter for 7 days has urine culture showing ≥10⁵ CFU/mL *Enterococcus* *faecalis*, but he has no fever, no flank pain, no suprapubic tenderness, and no systemic symptoms. | CAUTI | No | No qualification symptoms, NHSN cannot classify as CAUTI. | Standard | No | Correct | None |
| C12 |  | CAUTI | No |  | Structured | No | Correct | None |
| C12 |  | CAUTI | No |  | Constrained | No | Correct | None |
|  | | | | | | | | |
| C13 | A 64-year-old woman with a urinary catheter for 5 days develops fever. Urine culture grows *Candida* *albicans* (>10⁵ CFU/mL). | CAUTI | No | Yeasts do NOT meet NHSN CAUTI organism criteria. Fever alone + *Candida* = not CAUTI. | Standard | No | Correct | None |
| C13 |  | CAUTI | No |  | Structured | No | Correct | None |
| C13 |  | CAUTI | No |  | Constrained | No | Correct | None |
|  | | | | | | | | |
| C14 | A 71-year-old ICU patient has had a Foley catheter for 3 days. Develops fever (38.3°C) and costovertebral angle tenderness. Urine culture: ≥10⁵ CFU/mL *Klebsiella* *pneumoniae*. | CAUTI | YES - CAUTI | Eligible organism, symptoms meet criteria, catheter >2 days. | Standard | Yes | Correct | None |
| C14 |  | CAUTI | YES - CAUTI |  | Structured | Yes | Correct | None |
| C14 |  | CAUTI | YES - CAUTI |  | Constrained | Yes | Correct | None |
|  | | | | | | | | |
| C15 | A 50-year-old male had a Foley catheter removed yesterday (day 3 of admission). Today (day 4), he develops fever, and urine culture grows ≥10⁵ CFU/mL *Proteus* *mirabilis*. | CAUTI | No | For CAUTI classification, catheter must be in place for >2 days, AND in place on the date of event, OR removed within the previous 2 calendar days. Here the catheter was only in for 2 days total, does not qualify. | Standard | No | Correct | None |
| C15 |  | CAUTI | No |  | Structured | No | Correct | None |
| C15 |  | CAUTI | No |  | Constrained | No | Correct | None |
|  | | | | | | | | |
| C16 | A 62-year-old man with a Foley catheter in place for 6 days develops: fever (38.2°C), acute urinary urgency (new). Urine culture grows ≥10⁵ CFU/mL *Proteus* *mirabilis*. No other infection identified. | CAUTI | YES - CAUTI | Meets >2-day catheter requirement, symptomatic, organism eligible, no alternative source. | Standard | Yes | Correct | None |
| C16 |  | CAUTI | YES - CAUTI |  | Structured | Yes | Correct | None |
| C16 |  | CAUTI | YES - CAUTI |  | Constrained | Yes | Correct | None |
|  | | | | | | | | |
| C17 | A 70-year-old woman with an indwelling catheter for 4 days develops fever. Urine culture shows “mixed flora” with no predominant organism >10⁵ CFU/mL. | CAUTI | No | NHSN does not allow “mixed flora” or unspecified polymicrobial cultures for CAUTI classification. | Standard | No | Correct | None |
| C17 |  | CAUTI | No |  | Structured | No | Correct | None |
| C17 |  | CAUTI | No |  | Constrained | No | Correct | None |
|  | | | | | | | | |
| C18 | A 55-year-old male with a Foley catheter in place for 5 days develops fever and confusion. Urine culture: ≥10⁴ CFU/mL *E. coli* (below the required 10⁵ threshold). Chest X-ray shows left lower lobe pneumonia; sputum matches the BSI organism. | CAUTI | No | Urine does not meet threshold. Clear alternative source (pneumonia). Does NOT satisfy NHSN UTI criteria | Standard | No | Correct | None |
| C18 |  | CAUTI | No |  | Structured | No | Correct | None |
| C18 |  | CAUTI | No |  | Constrained | No | Correct | None |
|  | | | | | | | | |
| C19 | A 66-year-old woman had her urinary catheter present for 7 days, removed on day 8. On day 9, she develops fever and suprapubic tenderness. Urine culture: ≥10⁵ CFU/mL *Enterobacter* *cloacae*. | CAUTI | YES - CAUTI | NHSN rule: CAUTI can still be assigned if catheter removed ≤2 calendar days before date of event, and catheter had been in place >2 days. | Standard | Yes | Correct | None |
| C19 |  | CAUTI | YES - CAUTI |  | Structured | Yes | Correct | None |
| C19 |  | CAUTI | YES - CAUTI |  | Constrained | Yes | Correct | None |
|  | | | | | | | | |
| C20 | A 79-year-old ICU patient with a Foley for 10 days has routine urine testing. Culture grows ≥10⁵ CFU/mL Pseudomonas aeruginosa. The patient is sedated, afebrile, and has no new symptoms (NHSN allows only specific symptoms for CAUTI). | CAUTI | No | No qualifying symptoms, NHSN does not allow CAUTI classification. LLMs often mistakenly classify this as CAUTI because they assume any bacteriuria + catheter = CAUTI. | Standard | No | Correct | None |
| C20 |  | CAUTI | No |  | Structured | No | Correct | None |
| C20 |  | CAUTI | No |  | Constrained | No | Correct | None |
|  | | | | | | | | |
| C21 | A 68-year-old man develops ≥3 loose stools in 24 hours on hospital day 5. Stool toxin assay returns positive for toxigenic C. difficile. | CDI | YES - CDI | Hospital day ≥4, HO-CDI. Positive toxin + symptoms = meets criteria. | Standard | Yes | Correct | None |
| C21 |  | CDI | YES - CDI |  | Structured | Yes | Correct | None |
| C21 |  | CDI | YES - CDI |  | Constrained | Yes | Correct | None |
|  | | | | | | | | |
| C22 | A 72-year-old woman develops no GI symptoms but undergoes screening due to hospital outbreak. PCR for C. difficile is positive, toxin negative, and she has no diarrhoea. | CDI | No | NHSN requires diarrhoea (≥3 unformed stools in 24 hours). Asymptomatic PCR positivity = colonisation. | Standard | No | Correct | None |
| C22 |  | CDI | No |  | Structured | No | Correct | None |
| C22 |  | CDI | No |  | Constrained | No | Correct | None |
|  | | | | | | | | |
| C23 | A 55-year-old man was discharged from hospital 10 days ago. He now presents to the ED with profuse diarrhoea. Toxin test is positive. | CDI | YES - CDI | Symptomatic within ≤28 days of discharge, community-onset healthcare facility-associated. | Standard | Yes | Correct | None |
| C23 |  | CDI | YES - CDI |  | Structured | Yes | Correct | None |
| C23 |  | CDI | YES - CDI |  | Constrained | Yes | Correct | None |
|  | | | | | | | | |
| C24 | A 59-year-old woman with Crohn’s disease has chronic diarrhoea. Stool toxin is negative. PCR is positive. Clinicians believe this represents colonisation. | CDI | No | NHSN requires positive toxin OR NAAT + diarrhoea not explained by another cause. Here, diarrhoea is attributable to Crohn’s, does NOT count. | Standard | No | Correct | None |
| C24 |  | CDI | No |  | Structured | No | Correct | None |
| C24 |  | CDI | No |  | Constrained | No | Correct | None |
|  | | | | | | | | |
| C25 | A 63-year-old male had a CDI episode 4 weeks ago. Now has new diarrhoea (≥3 loose stools/24h) and a positive toxin test. | CDI | YES - CDI | Occurs within 2-8 weeks of previous episode, recurrent CDI per NHSN. | Standard | Yes | Correct | None |
| C25 |  | CDI | YES - CDI |  | Structured | Yes | Correct | None |
| C25 |  | CDI | YES - CDI |  | Constrained | Yes | Correct | None |
|  | | | | | | | | |
| C26 | A 78-year-old woman develops diarrhoea on hospital day 3 after receiving two doses of lactulose for constipation. Stool toxin is negative; NAAT (PCR) is positive. | CDI | No | Diarrhoea is fully explained by laxative use, which excludes CDI under NHSN rules. LLMs frequently misclassify this as CDI because of PCR positivity. | Standard | No | Correct | None |
| C26 |  | CDI | No |  | Structured | No | Correct | None |
| C26 |  | CDI | No |  | Constrained | No | Correct | None |
|  | | | | | | | | |
| C27 | A 70-year-old man received a single laxative dose 48 hours ago but now has ≥3 loose stools in 24 hours, abdominal pain, and leukocytosis. Stool toxin is positive for toxigenic C. difficile. | CDI | YES - CDI | A positive toxin overrides prior laxative exposure when the clinical picture fits. NHSN allows CDI classification if toxin is positive and symptoms are consistent. | Standard | Yes | Correct | None |
| C27 |  | CDI | YES - CDI |  | Structured | Yes | Correct | None |
| C27 |  | CDI | YES - CDI |  | Constrained | Yes | Correct | None |
|  | | | | | | | | |
| C28 | A 61-year-old man with no diarrhoea is screened using a multiplex stool PCR panel for an unrelated GI study. Panel detects toxigenic C. difficile DNA, but toxin is negative and no diarrhoea is present. | CDI | No | Asymptomatic + toxin negative, colonisation, not CDI. | Standard | No | Correct | None |
| C28 |  | CDI | No |  | Structured | No | Correct | None |
| C28 |  | CDI | No |  | Constrained | No | Correct | None |
|  | | | | | | | | |
| C29 | A 72-year-old woman on hospital day 2 has two loose stools, but no testing is done. On hospital day 4, she develops ≥3 loose stools, fever, and abdominal tenderness. Toxin assay is positive. | CDI | YES - CDI | Date of event = hospital day 4 (≥3 stools), toxin positive, hospital-onset CDI. NHSN does not count early isolated stools unless they meet the case definition. | Standard | Yes | Correct | None |
| C29 |  | CDI | YES - CDI |  | Structured | Yes | Correct | None |
| C29 |  | CDI | YES - CDI |  | Constrained | Yes | Correct | None |
|  | | | | | | | | |
| C30 | A patient had CDI treated 10 days ago (within the 2-week post-treatment window). Still has loose stools. Repeat PCR is positive, toxin negative. | CDI | No | Within the 14-day repeat testing exclusion window, NHSN does not allow a new CDI event to be reported. This is ongoing/persistent positivity, not a new episode. | Standard | No | Correct | None |
| C30 |  | CDI | No |  | Structured | No | Correct | None |
| C30 |  | CDI | No |  | Constrained | No | Correct | None |
|  | | | | | | | | |
| C31 | A 52-year-old woman undergoes a caesarean section. On postoperative day 6, she develops purulent drainage from the incision with surrounding erythema and tenderness. Wound swab grows *Staphylococcus* *aureus*. | SSI | YES | Purulent drainage within 30-day window from superficial incision, meets NHSN criteria. | Standard | Yes | Correct | None |
| C31 |  | SSI | YES |  | Structured | Yes | Correct | None |
| C31 |  | SSI | YES |  | Constrained | Yes | Correct | None |
|  | | | | | | | | |
| C32 | A 63-year-old man undergoes a hip replacement (implant present). On postoperative day 10, the wound is swollen with clear fluid. Orthopaedic team diagnoses seroma, not infection. Cultures are negative and no purulence. | SSI | No | Seroma without signs of infection does not meet superficial or deep SSI criteria. | Standard | No | Correct | None |
| C32 |  | SSI | No |  | Structured | No | Correct | None |
| C32 |  | SSI | No |  | Constrained | No | Correct | None |
|  | | | | | | | | |
| C33 | A 70-year-old woman undergoes colon surgery. On postoperative day 14, she develops fever and deep wound pain. The wound spontaneously opens, revealing purulent material beneath the fascia. Deep tissue culture grows *E. coli.* | SSI | YES - SSI | Purulence in deep soft tissues + matching organism + within 30 days. | Standard | Yes | Correct | None |
| C33 |  | SSI | YES - SSI |  | Structured | Yes | Correct | None |
| C33 |  | SSI | YES - SSI |  | Constrained | Yes | Correct | None |
|  | | | | | | | | |
| C34 | A 45-year-old man undergoes a routine appendectomy. On postoperative day 45, he develops redness and mild drainage from the incision. | SSI | No | Surveillance window is 30 days for this procedure, symptoms outside time window. | Standard | No | Correct | None |
| C34 |  | SSI | No |  | Structured | No | Correct | None |
| C34 |  | SSI | No |  | Constrained | No | Correct | None |
|  | | | | | | | | |
| C35 | A 67-year-old woman undergoes partial colectomy. On postoperative day 11, she develops fever and abdominal pain. CT scan shows an intra-abdominal abscess near the anastomosis. Drainage culture grows *Bacteroides* *fragilis*. | SSI | YES - SSI | Meets NHSN organ/space definition (intra-abdominal abscess + organism + within 30 days). | Standard | Yes | Correct | None |
| C35 |  | SSI | YES - SSI |  | Structured | Yes | Correct | None |
| C35 |  | SSI | YES - SSI |  | Constrained | Yes | Correct | None |
|  | | | | | | | | |
| C36 | A 58-year-old man undergoes lumbar spinal surgery with instrumentation (implant). On postoperative day 20, the incision is painful and swollen. Surgeon reopens the wound and finds purulent fluid beneath the fascia, but cultures remain negative. | SSI | YES - SSI | NHSN allows deep SSI without positive culture if clinical evidence of purulence beneath fascia is present. | Standard | Yes | Correct | None |
| C36 |  | SSI | YES - SSI |  | Structured | Yes | Correct | None |
| C36 |  | SSI | YES - SSI |  | Constrained | Yes | Correct | None |
|  | | | | | | | | |
| C37 | A 45-year-old woman undergoes breast reduction surgery. On day 7, the wound becomes swollen with dark bloody fluid. Surgeon evacuates a hematoma; cultures are negative, and there is no purulence or erythema. | SSI | No | Hematomas are non-infectious postoperative complications and do not meet SSI criteria. | Standard | No | Correct | None |
| C37 |  | SSI | No |  | Structured | No | Correct | None |
| C37 |  | SSI | No |  | Constrained | No | Correct | None |
|  | | | | | | | | |
| C38 | A 73-year-old man undergoes gastrectomy. On postoperative day 16: fever, abdominal pain, CT shows an abscess adjacent to the surgical site, consistent with an anastomotic leak. Drainage grows *Enterococcus* *faecalis*. | SSI | YES - SSI | Meets organ/space SSI definition: abscess in anatomical compartment opened/manipulated during surgery + organism. | Standard | Yes | Correct | None |
| C38 |  | SSI | YES - SSI |  | Structured | Yes | Correct | None |
| C38 |  | SSI | YES - SSI |  | Constrained | Yes | Correct | None |
|  | | | | | | | | |
| C39 | A 62-year-old woman had a knee prosthesis implanted. On postoperative day 110, she develops wound drainage and pain. Swab grows Staph epidermidis. | SSI | No | Implant surgeries have a 90-day SSI surveillance window, symptoms at day 110 are outside the NHSN window, regardless of culture. | Standard | No | Correct | None |
| C39 |  | SSI | No |  | Structured | No | Correct | None |
| C39 |  | SSI | No |  | Constrained | No | Correct | None |
|  | | | | | | | | |
| C40 | A 60-year-old man undergoes hernia repair. On postoperative day 5, he becomes febrile.  The incision is clean. Blood cultures grow *Streptococcus* *pneumoniae*, and chest X-ray confirms pneumonia. No wound symptoms or purulence. | SSI | No | Blood cultures grow *Streptococcus* *pneumoniae*, and chest X-ray confirms pneumonia. No wound symptoms or purulence. | Standard | No | Correct | None |
| C40 |  | SSI | No |  | Structured | No | Correct | None |
| C40 |  | SSI | No |  | Constrained | No | Correct | None |
|  | | | | | | | | |
| C41 | A 66-year-old man is mechanically ventilated for 5 days following surgery. On ventilator day 6, he develops: purulent tracheal secretions, fever (38.4°C), leukocytosis. CXR shows new right lower lobe infiltrate. Endotracheal aspirate culture grows Pseudomonas aeruginosa. | VAP | YES - VAP | New infiltrate + systemic signs + purulent secretions + qualifying organism, meets NHSN VAP criteria. | Standard | Yes | Correct | None |
| C41 |  | VAP | YES - VAP |  | Structured | Yes | Correct | None |
| C41 |  | VAP | YES - VAP |  | Constrained | Yes | Correct | None |
|  | | | | | | | | |
| C42 | A 54-year-old woman on a ventilator for 7 days develops fever and increased secretions. CXR: unchanged compared to prior imaging, no new infiltrate. Sputum grows *Klebsiella*, but radiology is unchanged. | VAP | No | NHSN requires a new/progressive infiltrate. Without radiologic change, cannot classify as VAP. | Standard | No | Correct | None |
| C42 |  | VAP | No |  | Structured | No | Correct | None |
| C42 |  | VAP | No |  | Constrained | No | Correct | None |
|  | | | | | | | | |
| C43 | A 70-year-old male ventilated for 4 days develops: fever, worsening oxygenation, obvious purulent secretions. CXR: new bilateral infiltrates. No respiratory culture collected. | VAP | YES - VAP | NHSN allows VAP classification without culture if: radiology shows new infiltrate, purulent secretions present, patient meets systemic criteria. This meets clinical VAP. | Standard | Yes | Correct | None |
| C43 |  | VAP | YES - VAP |  | Structured | Yes | Correct | None |
| C43 |  | VAP | YES - VAP |  | Constrained | Yes | Correct | None |
|  | | | | | | | | |
| C44 | A 60-year-old woman ventilated for 5 days develops low-grade fever. CXR shows basal atelectasis, confirmed by radiology as non-infectious. Cultures are negative. | VAP | No | Atelectasis is a common non-infectious finding and does not meet radiologic pneumonia criteria. | Standard | No | Correct | None |
| C44 |  | VAP | No |  | Structured | No | Correct | None |
| C44 |  | VAP | No |  | Constrained | No | Correct | None |
|  | | | | | | | | |
| C45 | A 73-year-old man on mechanical ventilation for 6 days develops fever and worsening oxygenation.  Bronchoalveolar lavage (BAL) culture grows Staph aureus at >10⁴ CFU/mL (threshold met).  CXR shows a new infiltrate. | VAP | YES - VAP | BAL >10⁴ CFU + new infiltrate + systemic signs meet VAP criteria with quantitative culture confirmation. | Standard | Yes | Correct | None |
| C45 |  | VAP | YES - VAP |  | Structured | Yes | Correct | None |
| C45 |  | VAP | YES - VAP |  | Constrained | Yes | Correct | None |
|  | | | | | | | | |
| C46 | A 75-year-old woman is ventilated for 8 days. She develops fever and worsening oxygenation. CXR shows bilateral opacities consistent with pulmonary oedema, confirmed by radiologist (cardiogenic pattern). BAL culture grows normal respiratory flora only. | VAP | No | Radiology shows pulmonary oedema, not pneumonia. NHSN explicitly requires infiltrates not attributable to non-infectious causes. | Standard | No | Correct | None |
| C46 |  | VAP | No |  | Structured | No | Correct | None |
| C46 |  | VAP | No |  | Constrained | No | Correct | None |
|  | | | | | | | | |
| C47 | A 62-year-old man ventilated for 5 days develops fever and purulent secretions. CXR sequence shows progressive right lower lobe infiltrate over 48 hours. Respiratory PCR is positive for influenza A, but sputum culture grows *Staph aureus*. | VAP | YES - VAP | Viral infection does not exclude VAP if a bacterial pathogen is present with radiologic progression and clinical signs. | Standard | Yes | Correct | None |
| C47 |  | VAP | YES - VAP |  | Structured | Yes | Correct | None |
| C47 |  | VAP | YES - VAP |  | Constrained | Yes | Correct | None |
|  | | | | | | | | |
| C48 | A 58-year-old man was mechanically ventilated for 3 days. Ventilator was removed 3 days ago. Today he develops fever, purulent sputum, and a new infiltrate on CXR. | VAP | No | For VAP classification, the event must occur on mechanical ventilation, OR within ≤2 calendar days after ventilator removal. Here symptoms appear day 3 post-extubation, outside NHSN window. | Standard | No | Correct | None |
| C48 |  | VAP | No |  | Structured | No | Correct | None |
| C48 |  | VAP | No |  | Constrained | No | Correct | None |
|  | | | | | | | | |
| C49 | A 69-year-old woman has been ventilated for 7 days. She develops fever, purulent secretions, and worsening oxygenation. CXR shows a new infiltrate. BAL culture grows *Klebsiella* *pneumoniae* at 10³ CFU/mL (below quantitative VAP threshold). | VAP | YES - VAP | Meets VAP criteria based on radiologic progression + purulent secretions + systemic signs. NHSN does not require a quantitative culture threshold for VAP. | Standard | Yes | Correct | None |
| C49 |  | VAP | YES - VAP |  | Structured | Yes | Correct | None |
| C49 |  | VAP | YES - VAP |  | Constrained | Yes | Correct | None |
|  | | | | | | | | |
| C50 | A 71-year-old ICU patient ventilated for 10 days has fever and purulent secretions. CXR is unchanged compared to prior imaging. Endotracheal aspirate culture grows coagulase-negative *Staphylococcus* in one specimen. | VAP | No | No new or progressive infiltrate cannot classify as VAP. Organism likely a contaminant. NHSN requires radiologic evidence as a mandatory component. | Standard | No | Correct | None |
| C50 |  | VAP | No |  | Structured | No | Correct | None |
| C50 |  | VAP | No |  | Constrained | No | Correct | None |
|  | | | | | | | | |
| C51 | A 67-year-old man with a central venous catheter in place for 5 days develops fever and hypotension on hospital day 7. Two blood culture sets grow *Klebsiella pneumoniae*. He has mild abdominal pain, but CT abdomen shows only diverticulosis with no clear evidence of diverticulitis or abscess. Urine culture is negative. No respiratory or urinary source is identified. | CLABSI | No | This does not meet criteria for a secondary BSI, but no alternative NHSN-defined primary site is established. Because the patient has a central line >2 days and a recognised pathogen in ≥2 blood cultures, this meets LCBI-1, CLABSI. Does NOT meet CLABSI criteria because the BSI is attributed as a secondary BSI to an intra-abdominal source based on abdominal symptoms and clinical diagnosis consistent with an IAB event. Therefore, it cannot be classified as CLABSI. | Standard | Yes | GPT incorrectly classified a secondary BSI as CLABSI | Misclassification, secondary BSI mislabelled as CLABSI |
| C51 |  | CLABSI | No |  | Structured | Yes | GPT incorrectly classified a secondary BSI as CLABSI | Misclassification, secondary BSI mislabelled as CLABSI |
| C51 |  | CLABSI | No |  | Constrained | Yes | GPT incorrectly classified a secondary BSI as CLABSI | Misclassification, secondary BSI mislabelled as CLABSI |
|  | | | | | | | | |
| C52 | A 72-year-old woman with an indwelling urinary catheter in place for 4 days develops new suprapubic tenderness and low-grade fever (38.1°C). Urinalysis shows pyuria. Urine culture grows 10³ CFU/mL *Pseudomonas aeruginosa*. No other infectious source is reported. | CAUTI | No | Urine culture <10⁵ CFU/mL; NHSN threshold not met for symptomatic CAUTI. | Standard | No | CFU count <10⁵ so CAUTI criteria not met | None |
| C52 |  | CAUTI | No |  | Structured | No | Correct | None |
| C52 |  | CAUTI | No |  | Constrained | No | Correct | None |
|  | | | | | | | | |
| C53 | A 59-year-old man develops diarrhoea (three loose stools in 24 hours) on hospital day 6 after receiving broad-spectrum antibiotics. Stool NAAT for *C. difficile* is positive, but toxin EIA is negative. No other pathogen is detected. He has no abdominal imaging findings. | CDI | No | NHSN requires a positive toxin assay or a positive multistep algorithm. NAAT+/toxin, does not meet CDI criteria. | Standard | Yes | GPT misclassified NAAT+/toxin, as CDI | Misclassification, did not apply toxin requirement |
| C53 |  | CDI | No |  | Structured | Yes | GPT misclassified NAAT+/toxin, as CDI | Misclassification, did not apply toxin requirement |
| C53 |  | CDI | No |  | Constrained | Yes | GPT misclassified NAAT+/toxin, as CDI | Misclassification, did not apply toxin requirement |
|  | | | | | | | | |
| C54 | A 48-year-old woman underwent abdominal hysterectomy 20 days ago. She now presents with fever and drainage from the incision. The wound shows erythema and tenderness. The surgeon expresses 5 mL of purulent fluid from a deeper pocket just beneath the fascia, but no imaging is performed. Culture grows *Streptococcus anginosus*. | SSI | YES - SSI | Deep incisional purulent drainage within 30 days of surgery meets NHSN deep-SSI criteria. | Standard | Yes | Deep incisional SSI criteria fully met | None |
| C54 |  | SSI | YES - SSI |  | Structured | Yes | Deep incisional SSI criteria fully met | None |
| C54 |  | SSI | YES - SSI |  | Constrained | Yes | Deep incisional SSI criteria fully met | None |
|  | | | | | | | | |
| C55 | A 66-year-old man has been mechanically ventilated for 6 days. He develops fever and increased oxygen requirement. Chest X-ray shows “patchy bilateral opacities possibly representing atelectasis or early pneumonia.” Endotracheal aspirate grows *Staphylococcus aureus*. Secretions are moderately purulent. | VAP | YES - VAP | New CXR opacities, worsening oxygenation, purulent secretions, and pathogen in ETA meet the VAC, IVAC, possible VAP pathway. | Standard | Yes | Meets the VAC, IVAC, possible VAP with acceptable radiology | None |
| C55 |  | VAP | YES - VAP |  | Structured | Yes | Meets the VAC, IVAC, possible VAP with acceptable radiology | None |
| C55 |  | VAP | YES - VAP |  | Constrained | Yes | Meets the VAC, IVAC, possible VAP with acceptable radiology | None |
|  | | | | | | | | |
| C56 | A 62-year-old man has had a central venous catheter in place for 6 days. He develops fever and hypotension. Two blood culture sets grow *Enterobacter* *cloacae*. He reports right upper quadrant abdominal pain. Ultrasound is inconclusive, showing “mild gallbladder wall thickening, no stones, no definite cholecystitis”. CT abdomen cannot be performed due to haemodynamic instability. Chest X-ray is normal. No urine culture is collected. | CLABSI | YES - CLABSI | No confirmed alternative source; central line >2 days and recognised pathogen in ≥2 blood cultures; equivocal abdominal imaging does not meet IAB criteria, so this is LCBI-1 CLABSI. | Standard | Yes | Correct | None |
| C56 |  | CLABSI | YES - CLABSI |  | Structured | Yes | Correct | None |
| C56 |  | CLABSI | YES - CLABSI |  | Constrained | Yes | Correct | None |
|  | | | | | | | | |
| C57 | A 71-year-old woman has had a Foley catheter in place for 3 days. She is afebrile but reports vague suprapubic discomfort. Urine culture grows 8 x 10⁴ CFU/mL *E. coli.* WBC count is normal. She also has a productive cough, and CXR shows “patchy basilar atelectasis versus early pneumonia”. No flank pain, no fever, no rigors. | CAUTI | No | Foley catheter >2 days and mild suprapubic discomfort but afebrile, urine culture 8×10⁴ CFU/mL *E. coli* (<10⁵) and possible respiratory source; CAUTI criteria not met. | Standard | Yes | GPT overcalled CAUTI despite CFU <10⁵ and limited symptoms | Threshold error, ignored CFU cut-off |
| C57 |  | CAUTI | No |  | Structured | No | Correct, after explicitly considering CFU threshold | None |
| C57 |  | CAUTI | No |  | Constrained | No | Correct, constrained prompt enforced CFU rule | None |
|  | | | | | | | | |
| C58 | A 67-year-old man develops three unformed stools in 24 hours on hospital day 5. He is receiving high dose magnesium supplements for hypomagnesaemia. Stool tests: NAAT positive, Toxin EIA negative No abdominal tenderness. No leukocytosis. Magnesium dosing increased the day before. | CDI | No | NAAT positive but toxin negative and diarrhoea plausibly explained by high dose magnesium; NHSN requires toxin positivity or unexplained compatible symptoms for CDI. | Standard | Yes | Equated NAAT positivity with CDI despite alternative cause | Definitional mismatch, ignored toxin and alternative explanation |
| C58 |  | CDI | No |  | Structured | Yes | Acknowledged magnesium but still classified as CDI | Overcalling CDI in NAAT+/toxin, context |
| C58 |  | CDI | No |  | Constrained | No | Correct, applied toxin requirement and alternative cause exclusion | None |
|  | | | | | | | | |
| C59 | A 45-year-old man undergoes laparoscopic appendectomy. On postoperative day 29, he develops low grade fever and reports mild incisional pain. Wound exam: erythema around the incision but no drainage or purulence. Ultrasound shows “small 1 cm subcutaneous fluid pocket, likely seroma versus early abscess”. No culture obtained. Symptoms resolve without antibiotics by day 31. | SSI | No | Postoperative day 29 after laparoscopic appendectomy with erythema and small likely seroma, no purulence, no culture, no fascial involvement; does not meet superficial, deep or organ/space SSI criteria. | Standard | Yes | Interpreted seroma and erythema as superficial SSI | Overcalling SSI, misclassified non-infectious seroma |
| C59 |  | SSI | No |  | Structured | No | Correct, distinguished seroma from SSI after stepwise reasoning | None |
| C59 |  | SSI | No |  | Constrained | No | Correct, constrained prompt emphasised purulence/culture requirements | None |
|  | | | | | | | | |
| C60 | A 68-year-old man is mechanically ventilated for 7 days. On ventilator day 8 he develops fever and mild increase in secretions. CXR report: “Diffuse interstitial markings that may represent pulmonary oedema, atelectasis, or early infection. No definite new consolidation.” BAL culture grows 1 x 10³ CFU/mL Pseudomonas aeruginosa. FiO₂ requirement increases from 30 percent to 40 percent. | VAP | No | Ventilated with fever and increased secretions but CXR shows no definite new or progressive infiltrate and BAL CFU below threshold; radiology suggests non-infectious causes, so VAP criteria are not met. | Standard | Yes | Classified clinically as VAP despite lack of definitive new infiltrate | Definition mismatch, did not require clear radiological progression |
| C60 |  | VAP | No |  | Structured | Yes | Recognised equivocal radiology but still labelled as VAP | Overcalling VAP in equivocal imaging |
| C60 |  | VAP | No |  | Constrained | No | Correct, required a definite new or progressive infiltrate and quantitative threshold | None |
|  | | | | | | | | |
| C61 | A 59-year-old neutropenic man (ANC 300) with a central line in place for 5 days develops fever and chills. Two blood culture sets grow *Enterococcus* *faecium*. He also has severe mucositis, with ulcerations throughout the oral cavity and oesophagus. Abdominal exam is benign. Chest X-ray is clear. No abdominal imaging was done. The team attributes his symptoms to mucosal damage from chemotherapy. | CLABSI | No | Neutropenia, severe mucositis and *Enterococcus* *faecium* (MBI organism) meet MBI-LCBI criteria, which are excluded from CLABSI counts under NHSN. | Standard | Yes | GPT incorrectly classified an MBI-LCBI as CLABSI | Misclassification, MBI-LCBI mislabelled as CLABSI |
| C61 |  | CLABSI | No |  | Structured | Yes | GPT incorrectly classified an MBI-LCBI as CLABSI | Misclassification, MBI-LCBI mislabelled as CLABSI |
| C61 |  | CLABSI | No |  | Constrained | Yes | GPT incorrectly classified an MBI-LCBI as CLABSI | Misclassification, MBI-LCBI mislabelled as CLABSI |
|  | | | | | | | | |
| C62 | A 64-year-old woman with a central line in place for 7 days develops fever and tachycardia. Two blood cultures grow *Streptococcus* *pneumoniae*. She also has a productive cough and CXR shows:  “Right lower lobe consolidation with air bronchograms.” Sputum culture grows S. pneumoniae as well. No abdominal or urinary symptoms. | CLABSI | No | *Streptococcus* *pneumoniae* BSI with matching organism and consolidation on CXR; meets pneumonia with secondary BSI so BSI is attributed to pneumonia, not CLABSI. | Standard | Yes | GPT assumed central line + BSI = CLABSI despite clear pneumonia | Incorrect source attribution, secondary BSI mislabelled as CLABSI |
| C62 |  | CLABSI | No |  | Structured | No | Correct, recognised pneumonia as primary site with secondary BSI | None |
| C62 |  | CLABSI | No |  | Constrained | No | Correct, applied NHSN secondary BSI hierarchy | None |
|  | | | | | | | | |
| C63 | A 55-year-old man had a CVC in place for 3 days, removed on hospital day 4. On hospital day 7 he develops fever. Two blood cultures grow *Klebsiella* *pneumoniae*. No alternative source is found. Central line has been out for 3 days. | CLABSI | No | Central line removed 3 days before date of event; exceeds ≤2-day device window for CLABSI attribution despite positive blood cultures and no confirmed alternative source. | Standard | Yes | Overlooked removal timing and treated as CLABSI | Timing rule error, ignored ≤2-day device window |
| C63 |  | CLABSI | No |  | Structured | Yes | Described chronology but still concluded CLABSI | Timing rule error, misapplied device-day logic |
| C63 |  | CLABSI | No |  | Constrained | No | Correct, constrained prompt enforced device removal window | None |
|  | | | | | | | | |
| C64 | A 72-year-old woman with a CVC develops fever. Blood cultures show one of two bottles positive for coagulase-negative *Staphylococcus*. Repeat cultures remain negative. No purulence at line site. No alternative site of infection identified. | CLABSI | No | Single positive bottle with coagulase negative *staphylococcus* and repeat cultures negative; meets contaminant definition and does not fulfil LCBI criteria. | Standard | Yes | Called CLABSI on a single CNS bottle | Threshold error, did not require ≥2 positive cultures |
| C64 |  | CLABSI | No |  | Structured | No | Correct, recognised CNS as contaminant in absence of repeat positivity | None |
| C64 |  | CLABSI | No |  | Constrained | No | Correct, constrained rules emphasised contaminant criteria | None |
|  | | | | | | | | |
| C65 | A 77-year-old woman with a Foley catheter for 4 days develops fever and suprapubic tenderness. Urine culture grows 4 x 10⁴ CFU/mL *E. coli.* Blood cultures are negative. No respiratory or abdominal findings. | CAUTI | No | Foley catheter >2 days with fever and suprapubic tenderness but urine culture 4×10⁴ CFU/mL (<10⁵) so quantitative threshold for CAUTI is not met. | Standard | Yes | Assumed any bacteriuria + catheter + symptoms = CAUTI | Threshold error, ignored 10⁵ CFU requirement |
| C65 |  | CAUTI | No |  | Structured | No | Correct, explicitly linked decision to CFU threshold | None |
| C65 |  | CAUTI | No |  | Constrained | No | Correct, rule-based prompt prevented CAUTI overcall | None |
|  | | | | | | | | |
| C66 | A 69-year-old man with a Foley in place for 3 days develops fever and altered mental status. Urinalysis shows pyuria. Urine culture grows mixed flora. CXR shows “new left lower lobe infiltrate.”  WBC elevated. | CAUTI | No | Mixed flora urine culture is not eligible for CAUTI and concurrent new infiltrate on CXR indicates pneumonia as the primary site of infection. | Standard | Yes | Labelled CAUTI based on pyuria and bacteriuria despite mixed flora and pneumonia | Definition/organism error, used ineligible culture and ignored alternative source |
| C66 |  | CAUTI | No |  | Structured | No | Correct, identified mixed flora as ineligible and recognised pneumonia | None |
| C66 |  | CAUTI | No |  | Constrained | No | Correct, constrained prompt enforced organism eligibility and alternative source | None |
|  | | | | | | | | |
| C67 | A 71-year-old man on tube feeds develops two loose stools on HD5. NAAT positive, toxin negative.  Tube feed rate was increased the day before. No abdominal pain or fever. | CDI | No | NAAT positive, toxin negative, only two loose stools and alternative explanation from enteral feeds; does not meet diarrhoea threshold or unexplained symptom requirement for CDI. | Standard | Yes | Interpreted NAAT positivity as CDI despite insufficient stools and clear alternative cause | Overcalling CDI, did not enforce stool count or alternative cause exclusion |
| C67 |  | CDI | No |  | Structured | Yes | Described feed related diarrhoea but still concluded CDI | Definitional drift, PCR driven classification |
| C67 |  | CDI | No |  | Constrained | No | Correct, applied stool frequency and alternative explanation rules | None |
|  | | | | | | | | |
| C68 | A 62-year-old woman receives high dose lactulose for hepatic encephalopathy. She has 5 loose stools within 24 hours. Stool toxin is positive. Laxative dosing occurred 3 hours before onset of symptoms. | CDI | YES - CDI | Patient has ≥3 loose stools in 24 hours and toxin assay is positive; NHSN allows CDI assignment when toxin is positive even in the context of recent laxative use. | Standard | No | Incorrectly dismissed CDI because of laxative use despite toxin positivity | Misinterpretation of NHSN, over-weighted alternative cause against toxin |
| C68 |  | CDI | YES - CDI |  | Structured | Yes | Correct, recognised that toxin positive meets CDI criteria despite laxatives | None |
| C68 |  | CDI | YES - CDI |  | Constrained | Yes | Correct, constrained prompt prioritised toxin rule as per NHSN | None |
|  | | | | | | | | |
| C69 | A 70-year-old man was ventilated for 10 days, extubated on day 11. On day 14 he develops fever, increased sputum, and new infiltrate. CXR shows: “New right upper lobe consolidation consistent with pneumonia.” Endotracheal aspirate grows Pseudomonas aeruginosa. | VAP | No | Pneumonia developing 3 days after extubation; exceeds the ≤2 calendar day ventilator window so this is not classified as VAP under NHSN. | Standard | Yes | Classified as VAP on clinical grounds without checking ventilator timing | Timing rule error, ignored post-extubation window |
| C69 |  | VAP | No |  | Structured | Yes | Described extubation timing but still labelled VAP | Timing logic failure, misapplied VAP window |
| C69 |  | VAP | No |  | Constrained | No | Correct, constrained rules enforced the ≤2-day post-extubation criterion | None |
|  | | | | | | | | |
| C70 | A 73-year-old man has been in the ICU for 9 days with a central venous catheter (in situ for all 9 days). He develops fever and hypotension on ICU Day 10. Findings: Two peripheral blood culture sets grow *Enterococcus* *faecalis*. He also has a Foley catheter in place for 5 days Urinalysis: pyuria. Urine culture: 10⁵ CFU/mL *Enterococcus* *faecalis*. No abdominal symptoms. Chest X-ray is clear. No mucositis, no neutropenia. No imaging of abdomen performed. Clinicians initially suspect CLABSI because the line looks clean and the patient is septic | CLABSI | No | Meets CAUTI criteria (indwelling catheter >2 days, fever, pyuria, urine ≥10⁵ CFU/mL *Enterococcus* *faecalis*) with matching organism in blood; BSI is secondary to CAUTI and therefore not a CLABSI. | Standard | No | Correct, GPT assigned CAUTI with secondary BSI and excluded CLABSI | None |
| C70 |  | CLABSI | No |  | Structured | No | Correct, stepwise reasoning followed NHSN secondary BSI hierarchy | None |
| C70 |  | CLABSI | No |  | Constrained | No | Correct, constrained prompt enforced CAUTI with secondary BSI, not CLABSI | None |
